# Supplementary material for: Marine fishes exhibit exceptional variation in biofluorescent emission spectra
Source: PLoS One. 2025 Jun 16;20(6):e0316789. doi: 10.1371/journal.pone.0316789 (PMC12169565; doi:10.1371/journal.pone.0316789)
Supplement: S2 Fig — (PDF) [file pone.0316789.s002.pdf]

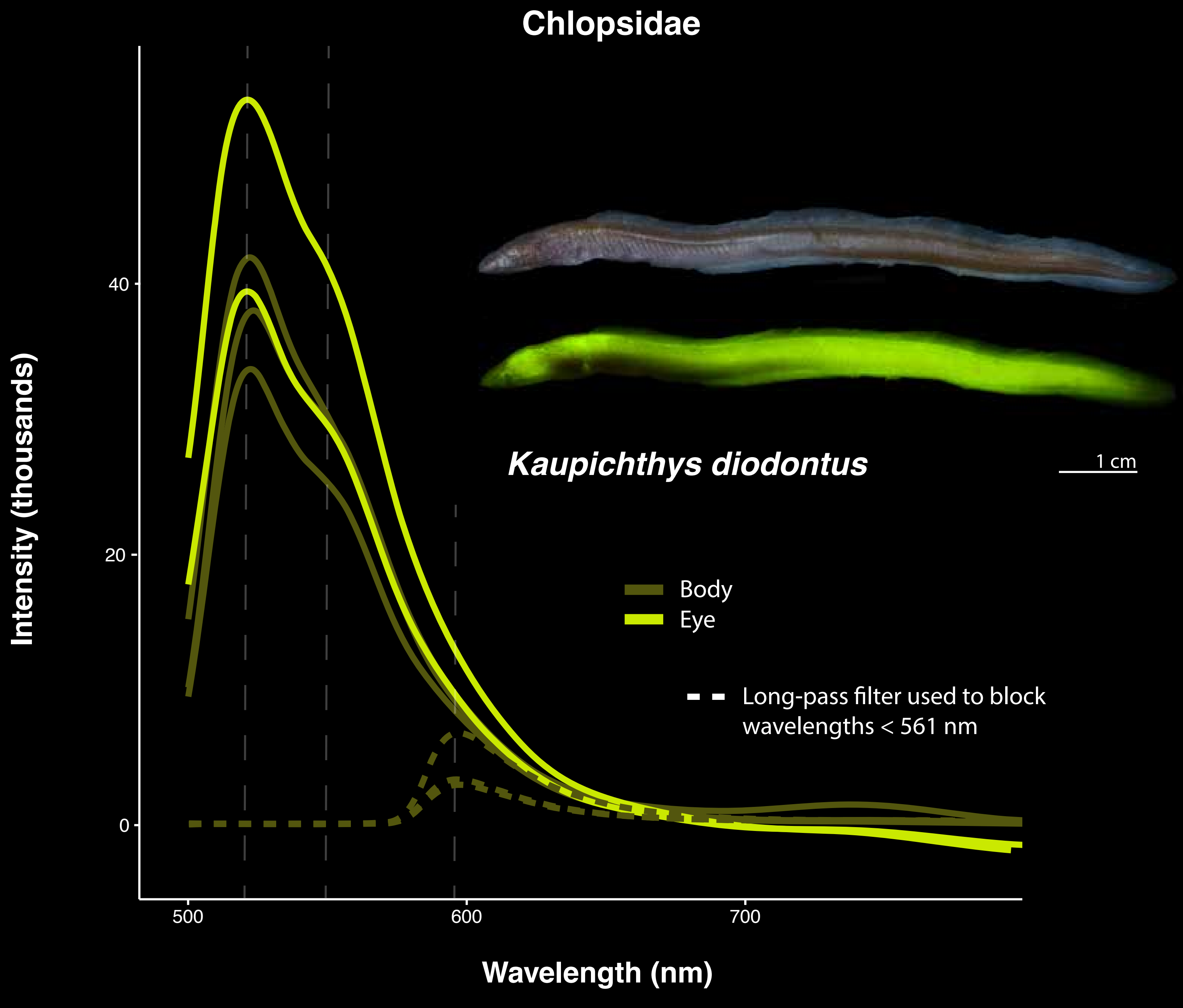
Chlopsidae
Intensity (thousands)
40
20
0
500
600
700
Wavelength (nm)
Kaupichthys diodontus
1 cm
Body
Eye
Long-pass filter used to block wavelengths < 561 nm

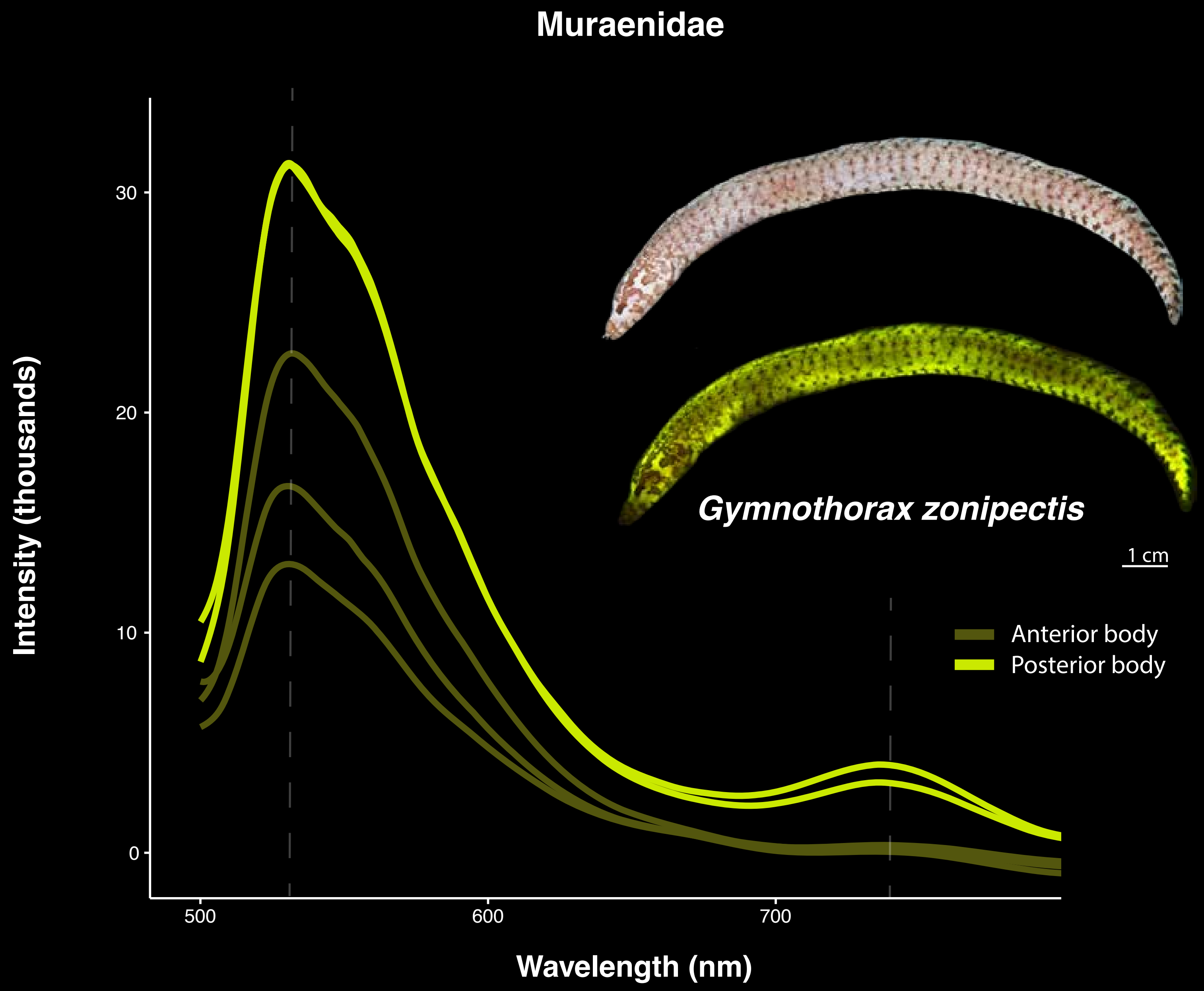

Muraenidae
Intensity (thousands)
30
20
10
0
500
600
700
Wavelength (nm)
Gymnothorax zonipectis
1 cm
Anterior body
Posterior body

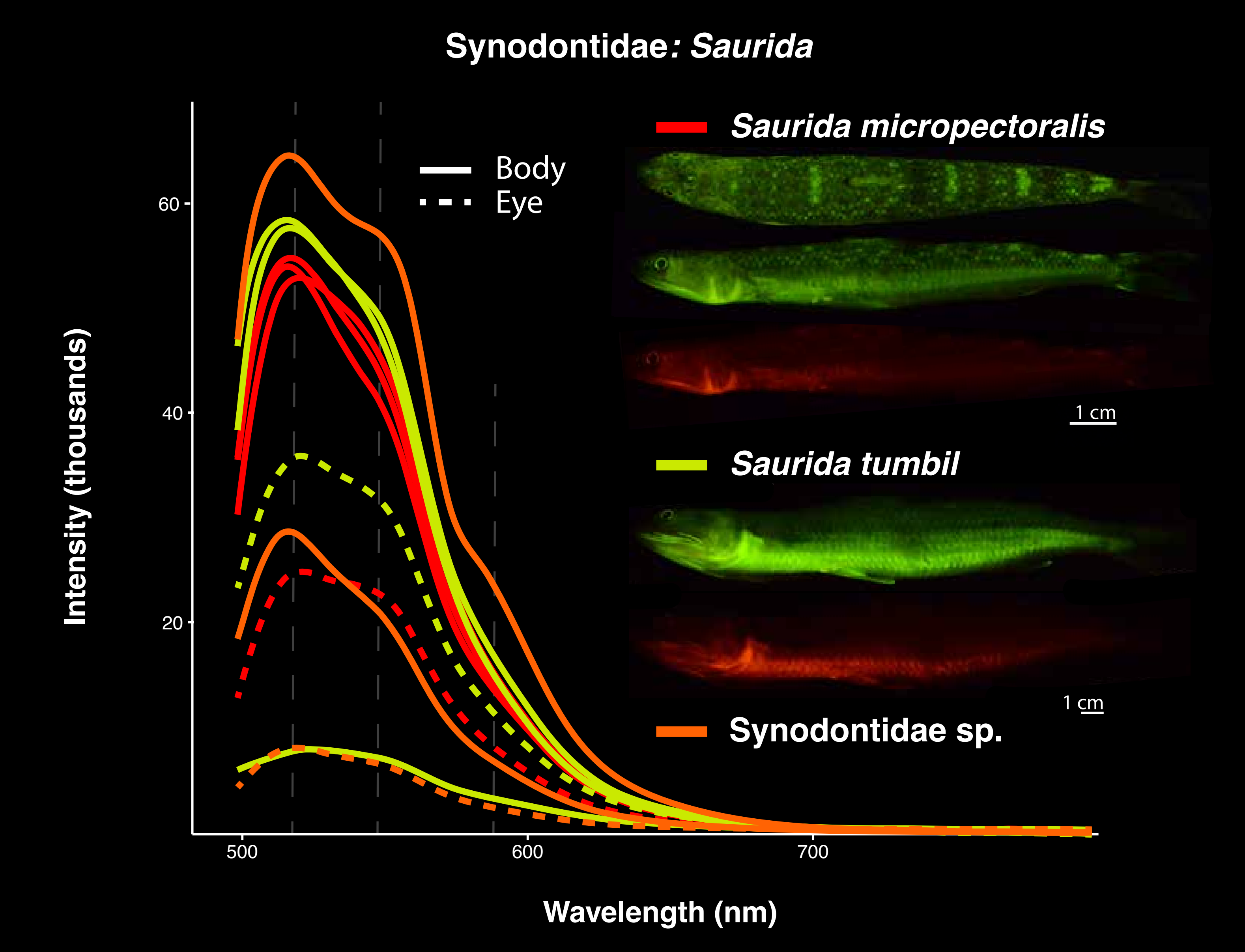

Synodontidae: Saurida
Intensity (thousands)
60
40
20
500
600
700
Wavelength (nm)
Body
Eye
Saurida micropectoralis
1 cm
Saurida tumbil
1 cm
Synodontidae sp.

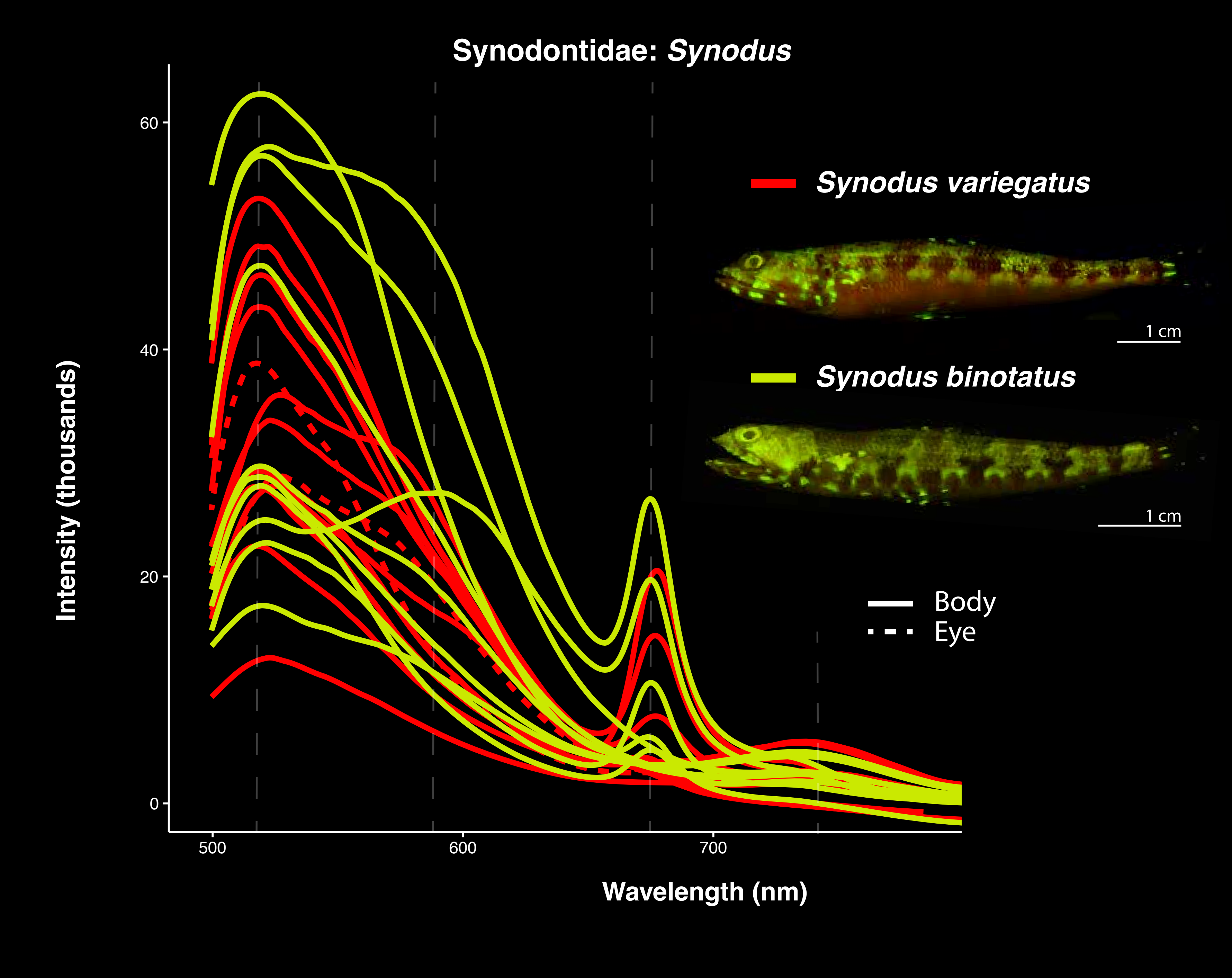

Synodontidae: *Synodus*
Synodus variegatus
Synodus binotatus
1 cm
1 cm
Body
Eye
Intensity (thousands)
0
20
40
60
500
600
700
Wavelength (nm)

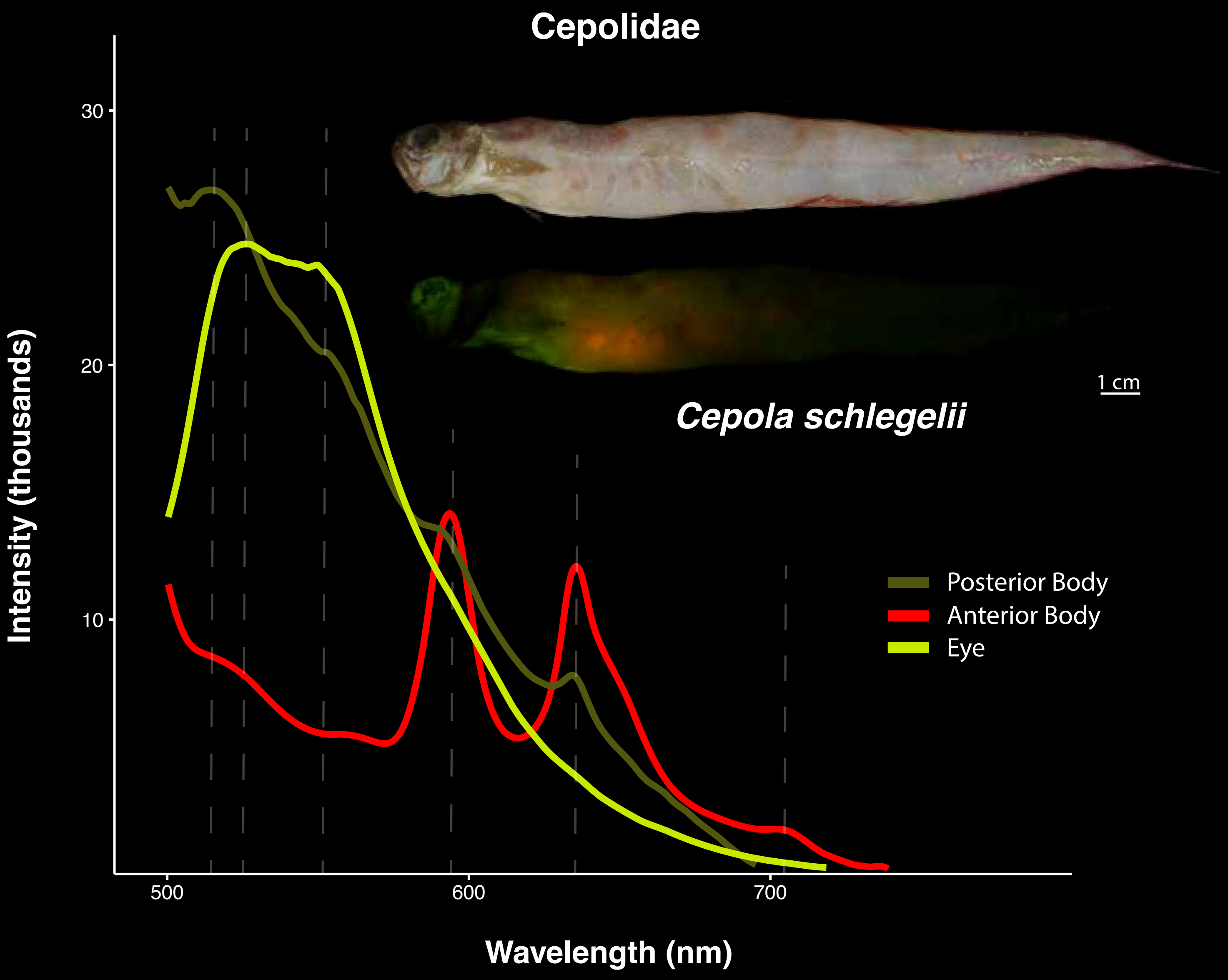

Cepolidae
Cepola schlegelii
1 cm
Intensity (thousands)
30
20
10
500
600
700
Wavelength (nm)
Posterior Body
Anterior Body
Eye

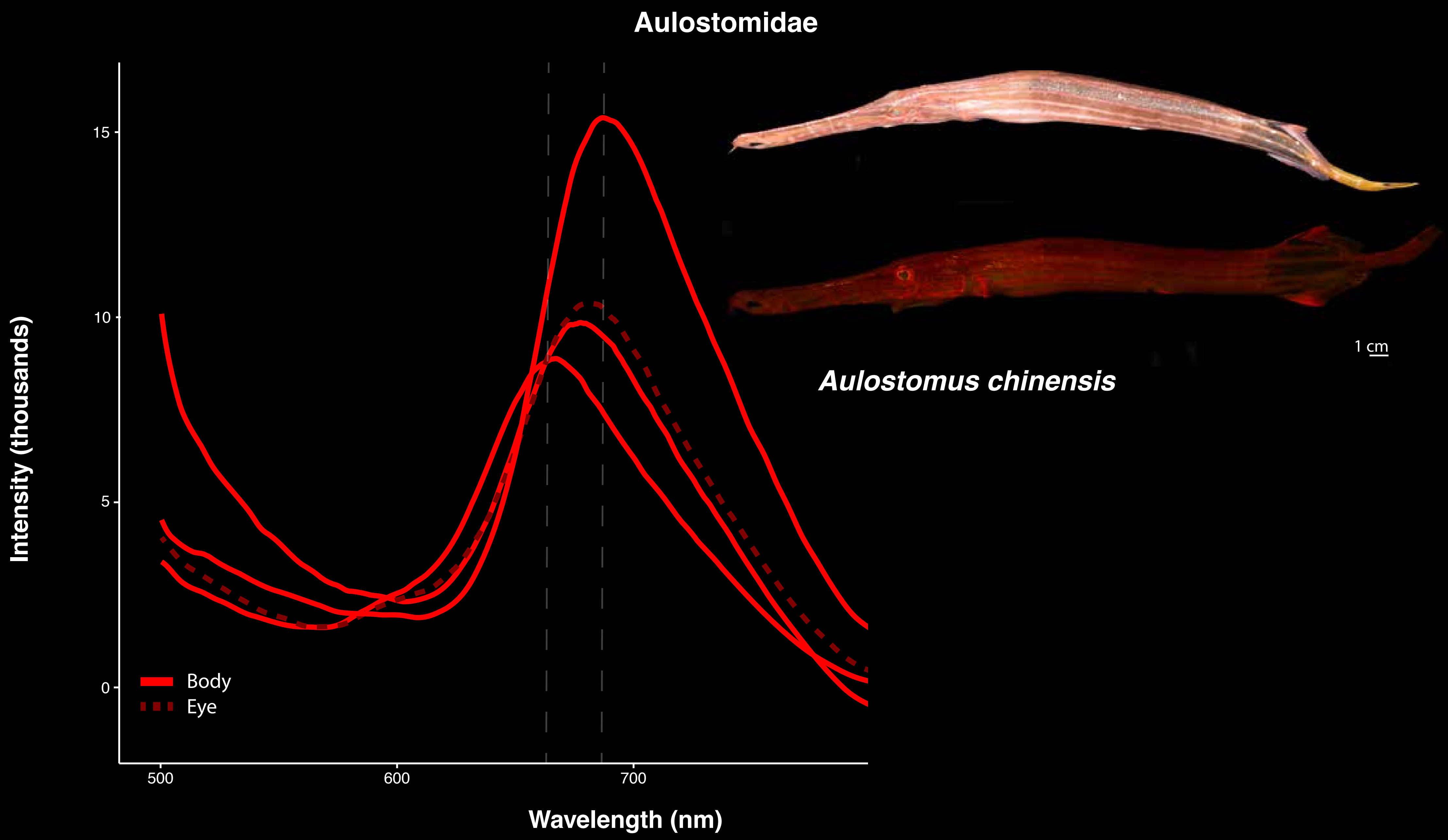

Aulostomidae
Intensity (thousands)
15
10
5
0
500
600
700
Wavelength (nm)
Body
Eye
1 cm
Aulostomus chinensis

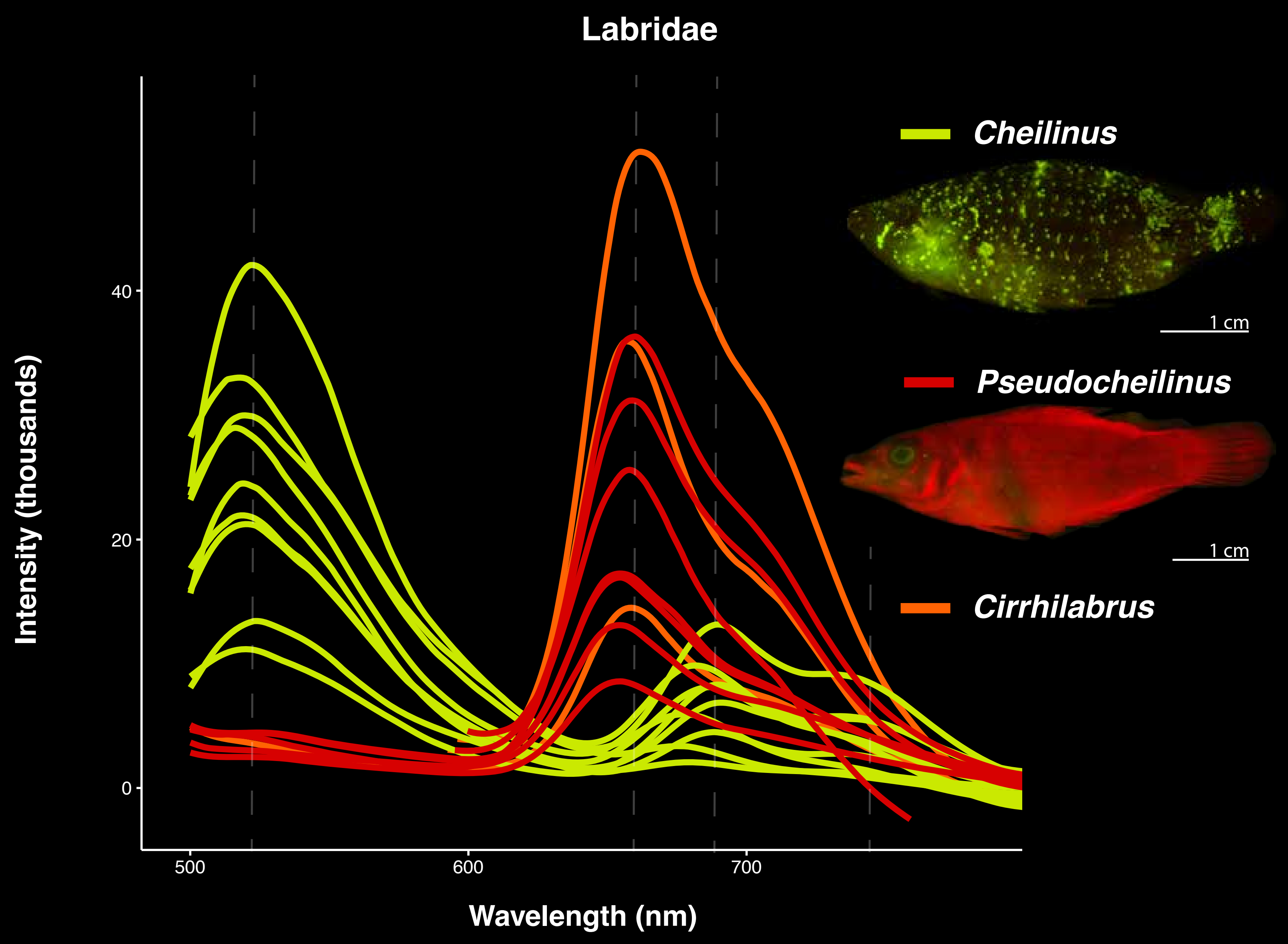

Labridae
Cheilinus
1 cm
Pseudocheilinus
1 cm
Cirrhilabrus
40
20
0
Intensity (thousands)
500
600
700
Wavelength (nm)

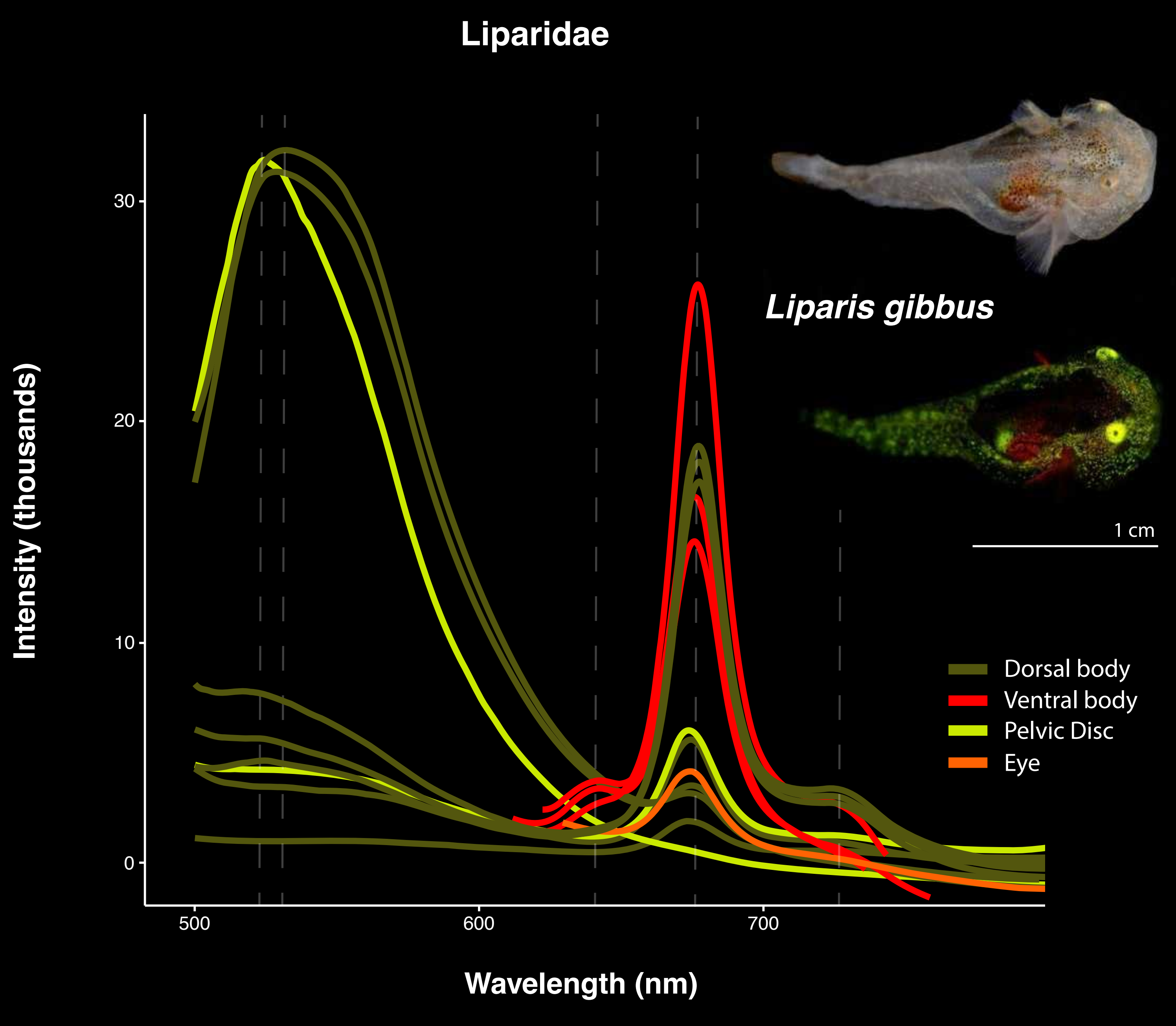

Liparidae
Liparis gibbus
1 cm
Intensity (thousands)
30
20
10
0
500
600
700
Wavelength (nm)
Dorsal body
Ventral body
Pelvic Disc
Eye

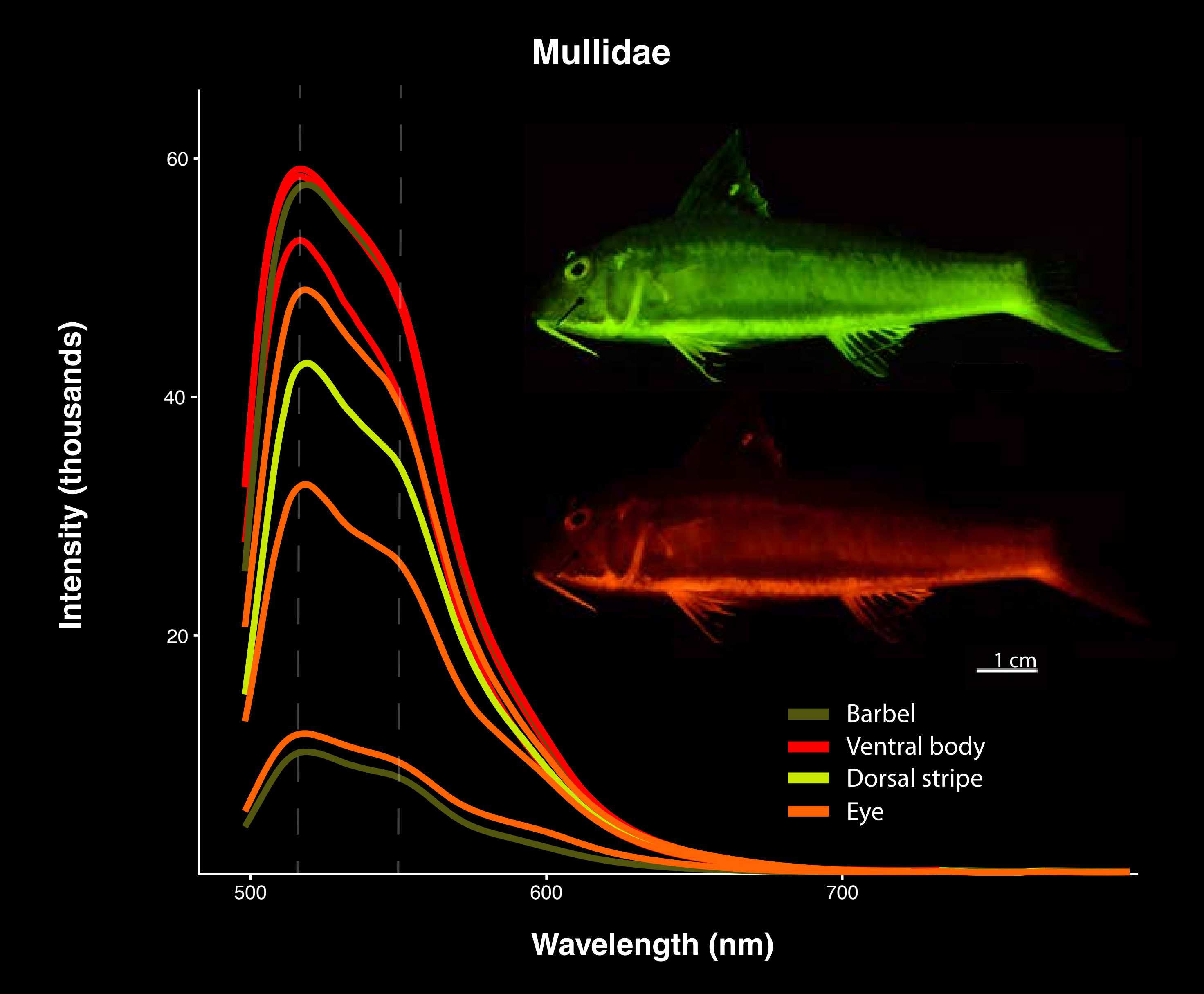

Mullidae
Intensity (thousands)
60
40
20
500
600
700
Wavelength (nm)
1 cm
Barbel
Ventral body
Dorsal stripe
Eye

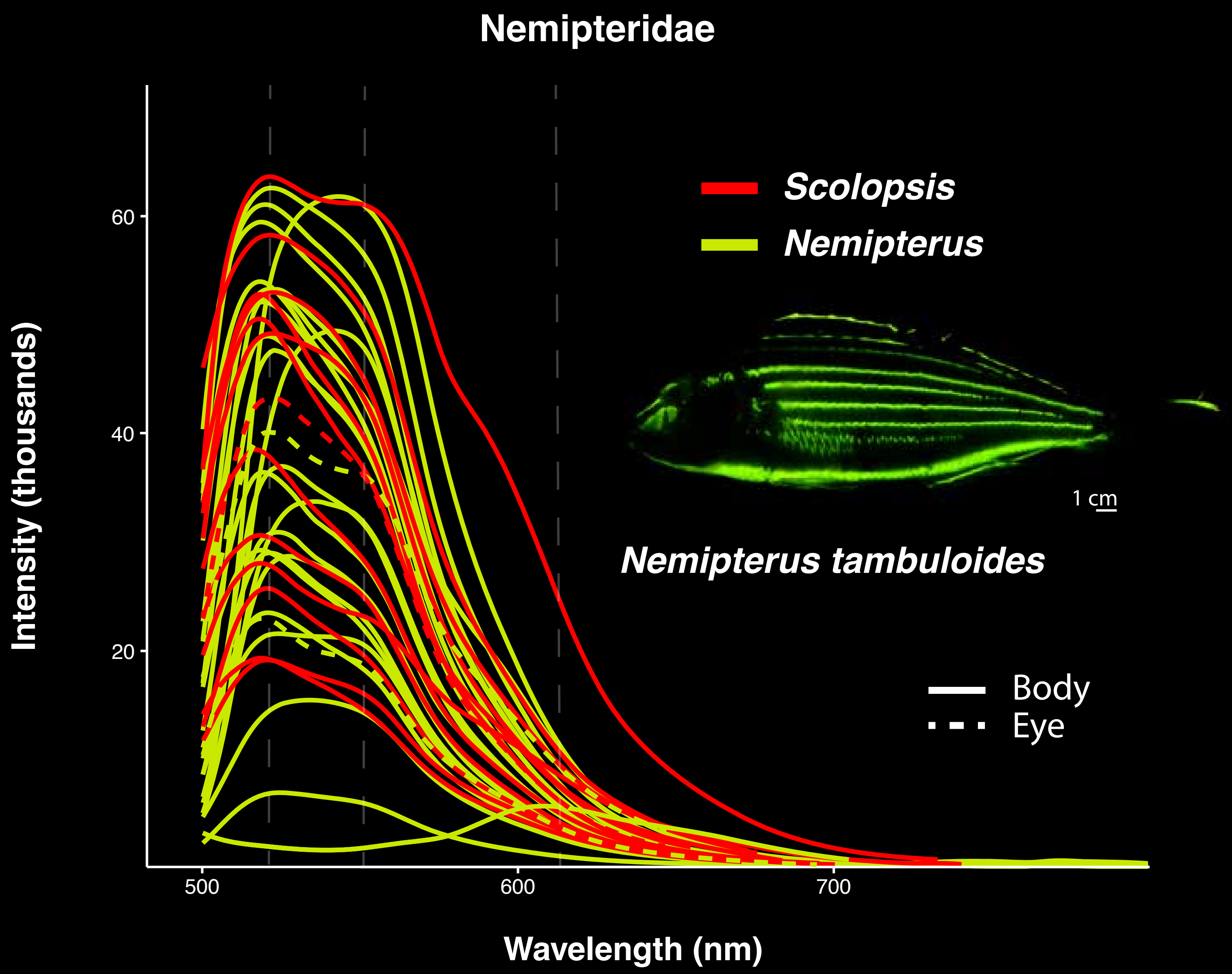

Nemipteridae
Intensity (thousands)
60
40
20
500
600
700
Wavelength (nm)
Scolopsis
Nemipterus
1 cm
Nemipterus tambuloides
Body
Eye

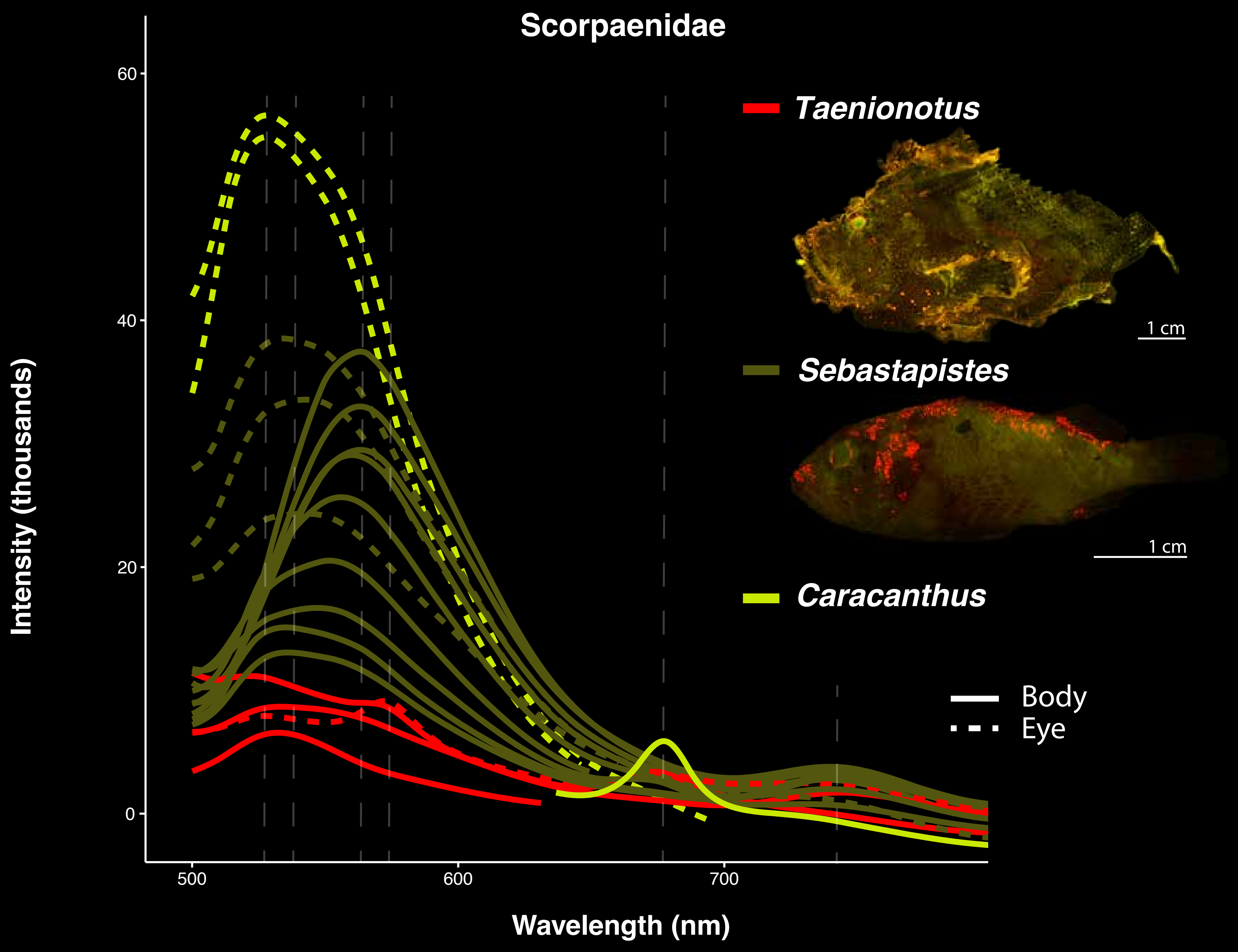
Scorpaenidae
Taenionotus
Sebastapistes
Caracanthus
1 cm
1 cm
Body
Eye
Intensity (thousands)
Wavelength (nm)
0
20
40
60
500
600
700

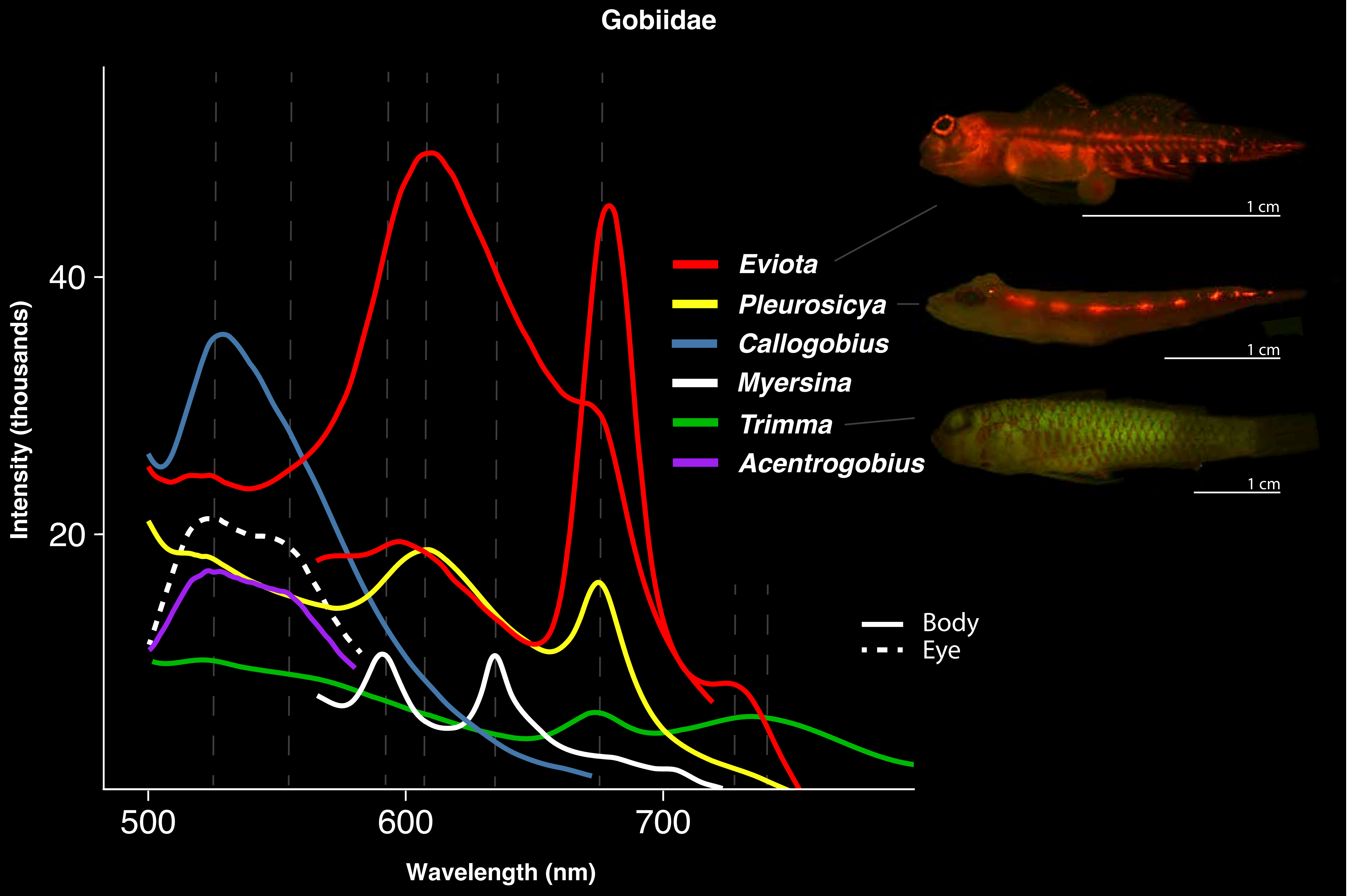

Gobiidae
Intensity (thousands)
40
20
500
600
700
Wavelength (nm)
Eviota
Pleurosicya
Callogobius
Myersina
Trimma
Acentrogobius
Body
Eye
1 cm
1 cm
1 cm

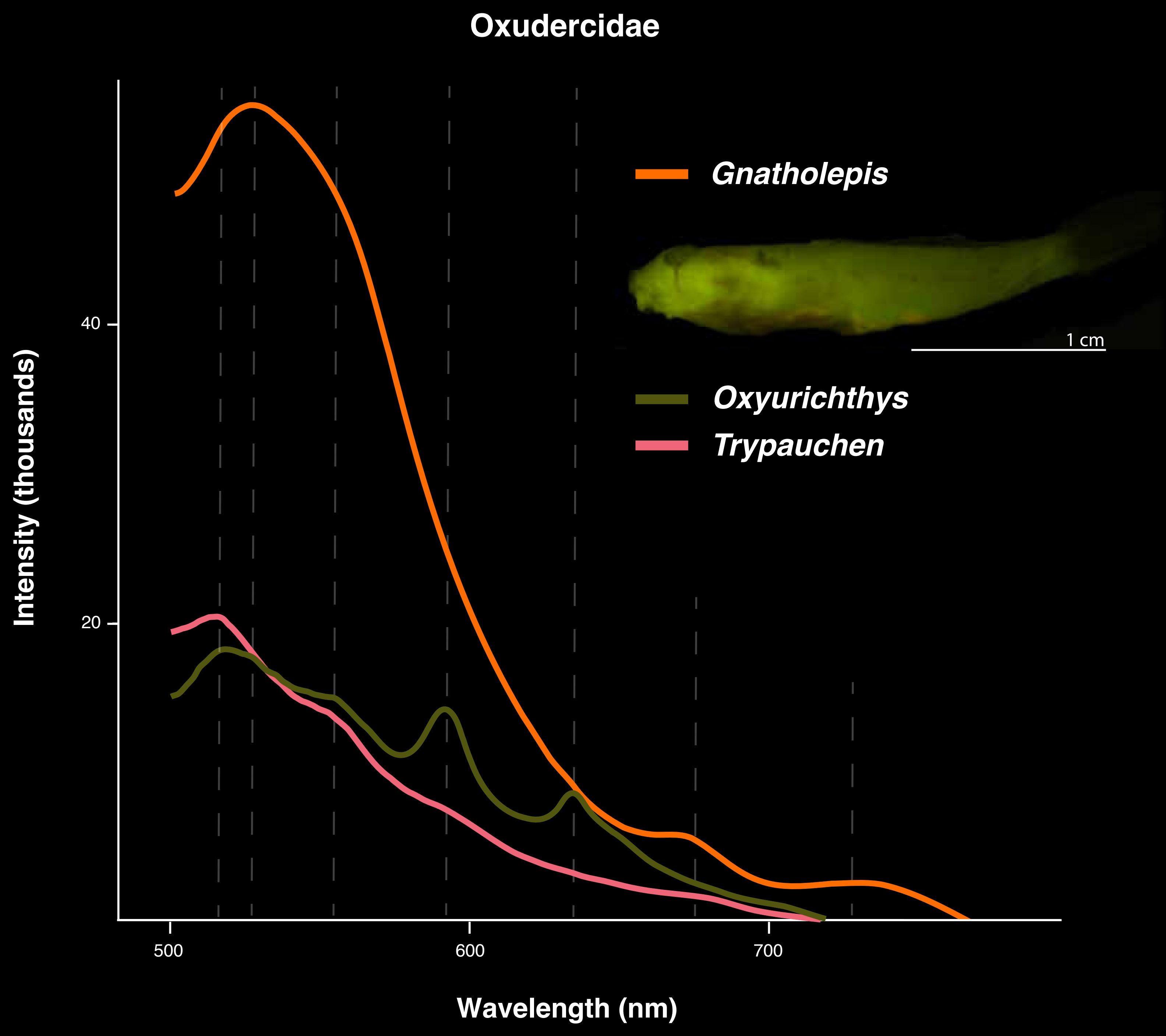
Oxudercidae
Gnatholepis
Oxyurichthys
Trypauchen
1 cm
40
20
500
600
700
Intensity (thousands)
Wavelength (nm)

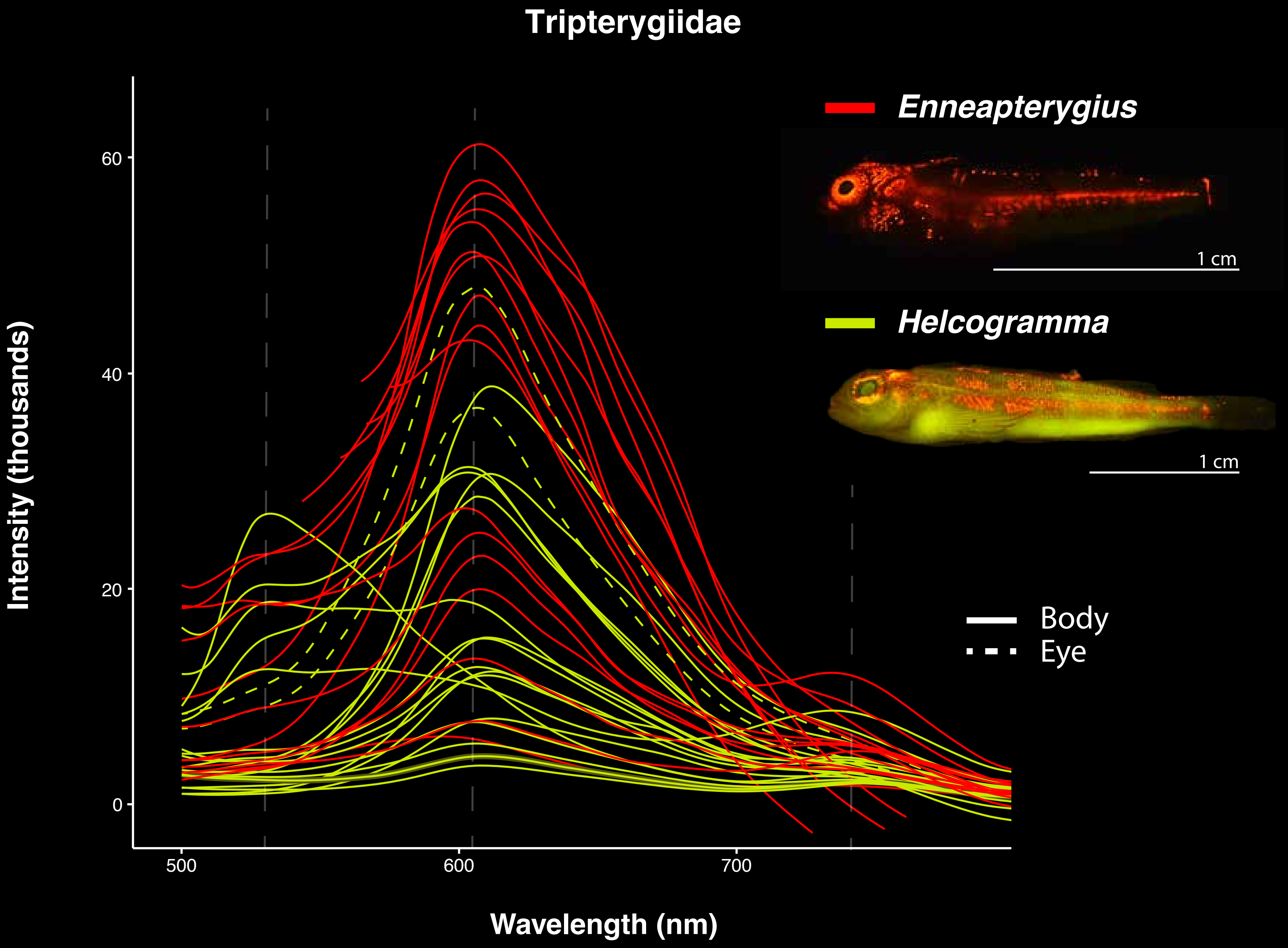
Tripterygiidae
Enneapterygius
1 cm
Helcogramma
1 cm
Body
Eye
Intensity (thousands)
60
40
20
0
500
600
700
Wavelength (nm)

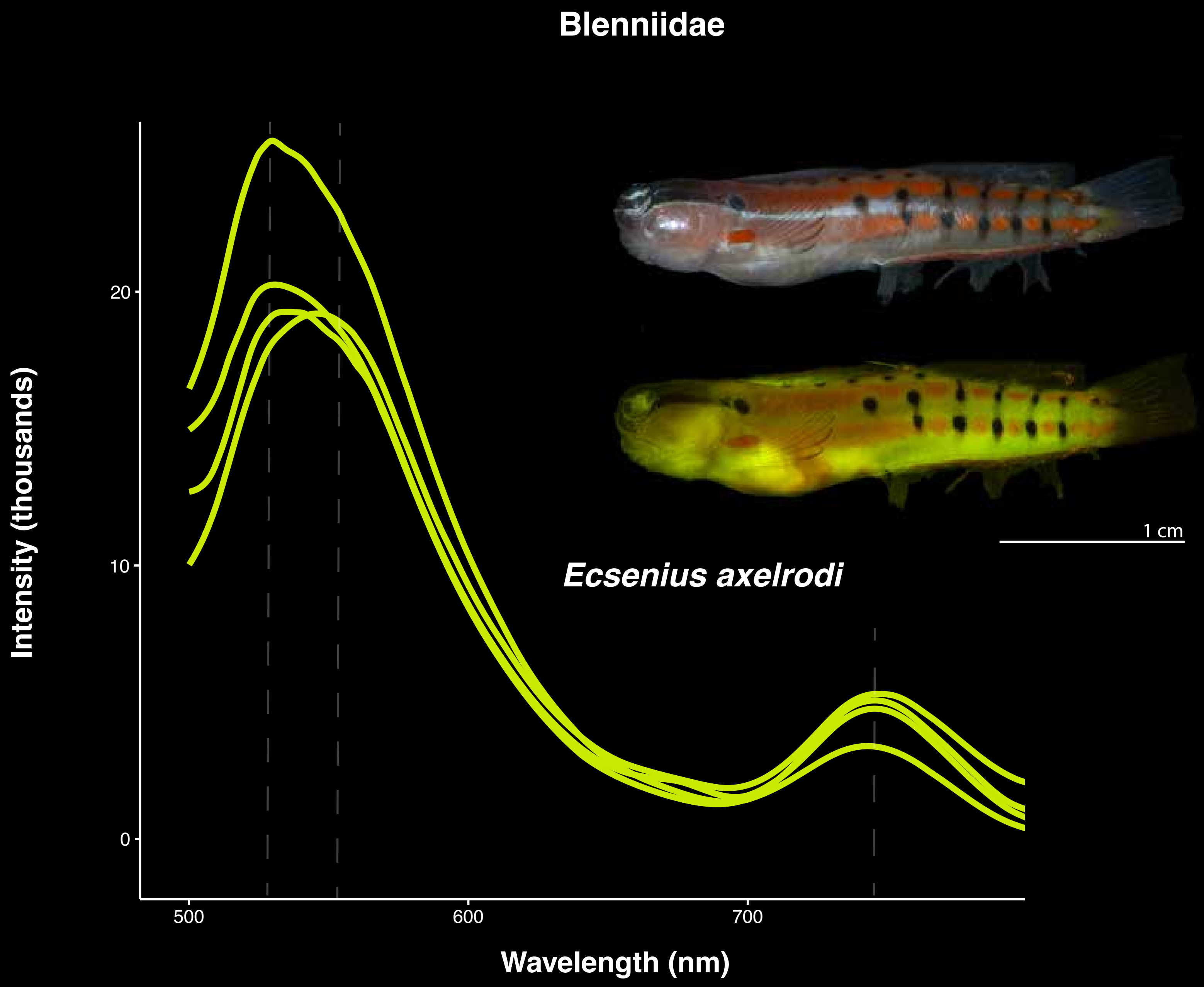

Blenniidae
Intensity (thousands)
20
10
0
500
600
700
Wavelength (nm)
Ecsenius axelrodi
1 cm

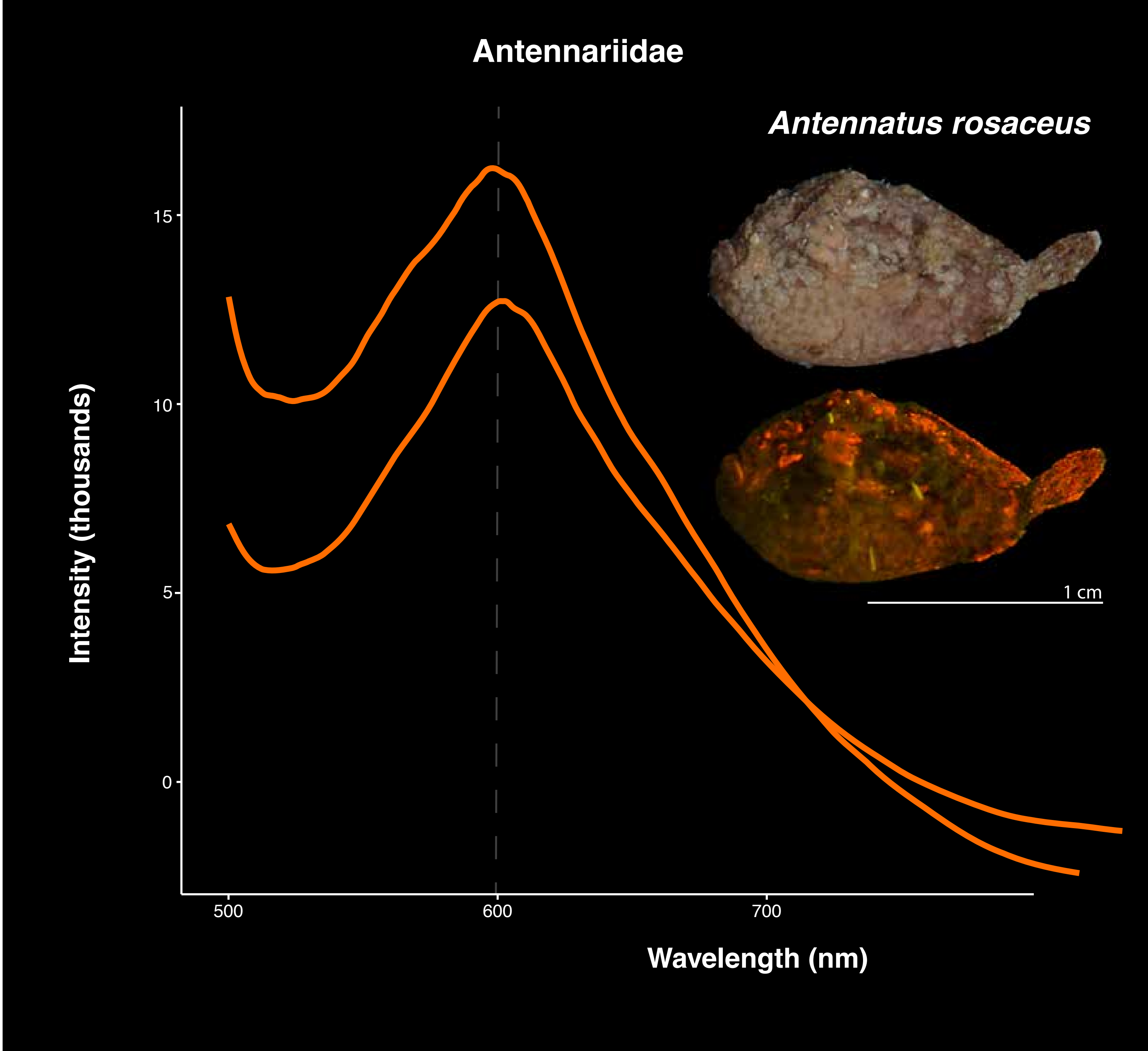

Antennariidae
Antennatus rosaceus
Intensity (thousands)
15
10
5
0
500
600
700
Wavelength (nm)
1 cm

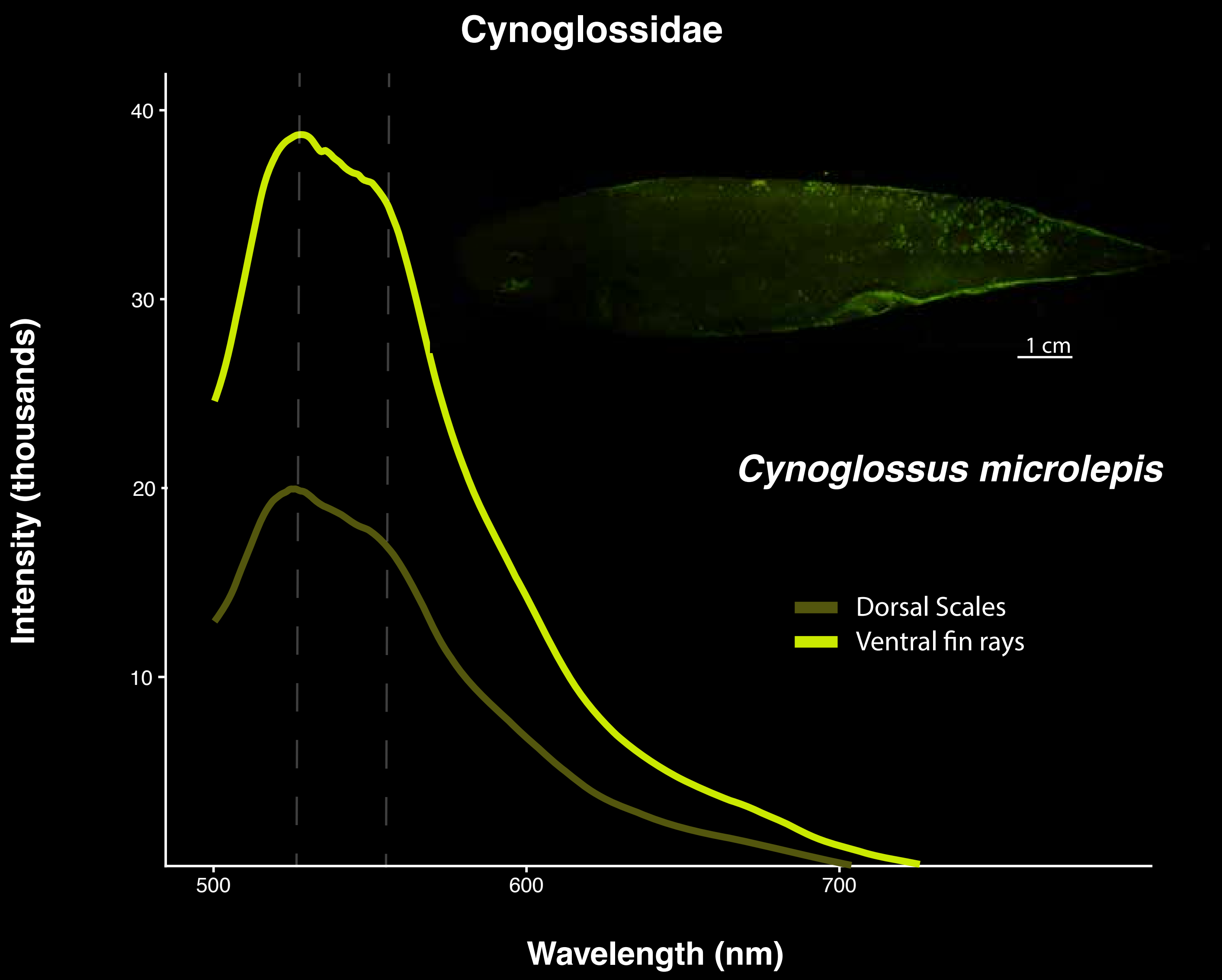

Cynoglossidae
40
30
20
10
Intensity (thousands)
500
600
700
Wavelength (nm)
1 cm
Cynoglossus microlepis
Dorsal Scales
Ventral fin rays

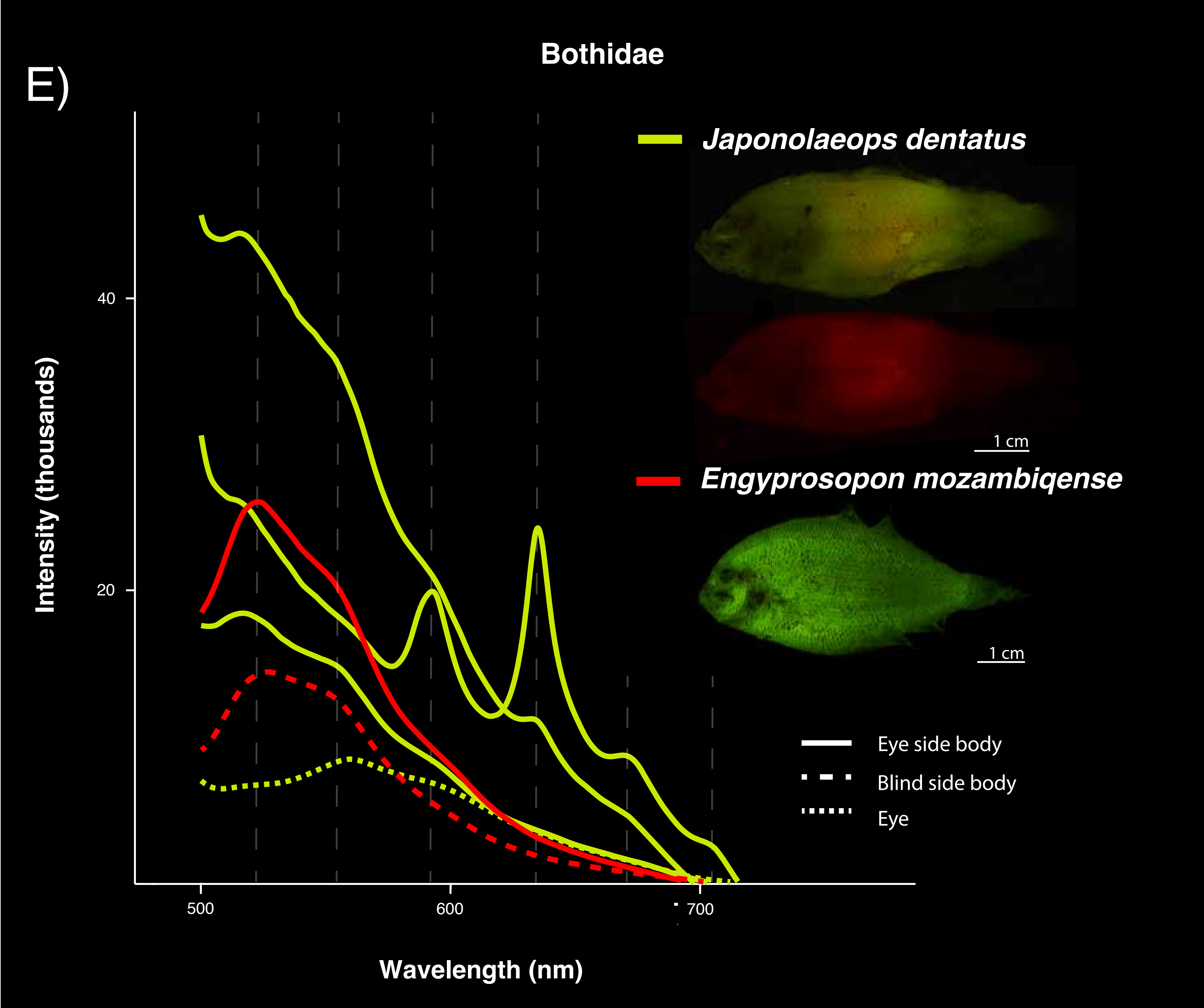
E)
Bothidae
Japonolaeops dentatus
1 cm
Engyprosopon mozambiqense
1 cm
Eye side body
Blind side body
Eye
Intensity (thousands)
40
20
500
600
700
Wavelength (nm)

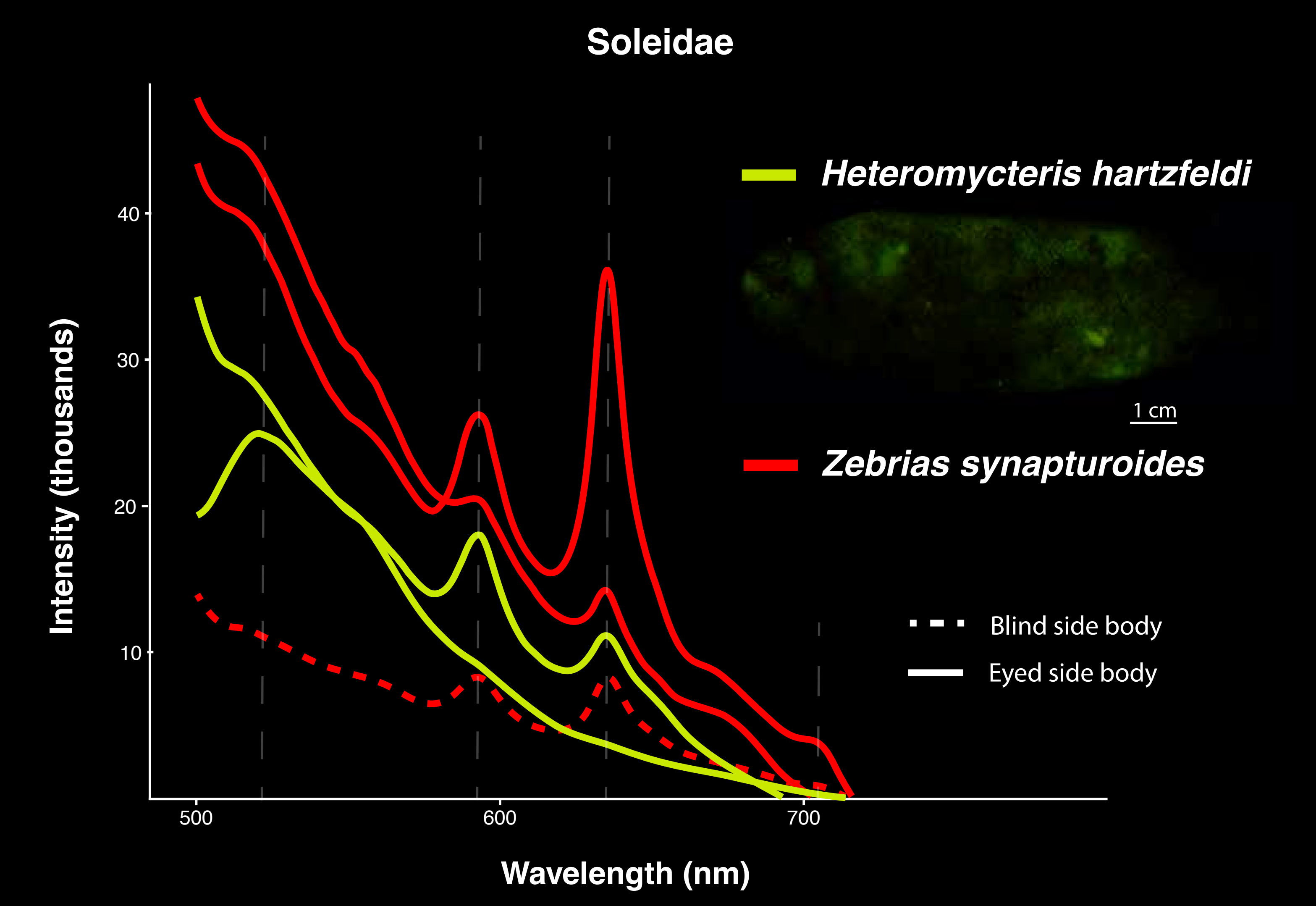

Soleidae
Heteromycteris hartzfeldi
Zebrias synapturoides
1 cm
Blind side body
Eyed side body
Intensity (thousands)
10
20
30
40
500
600
700
Wavelength (nm)

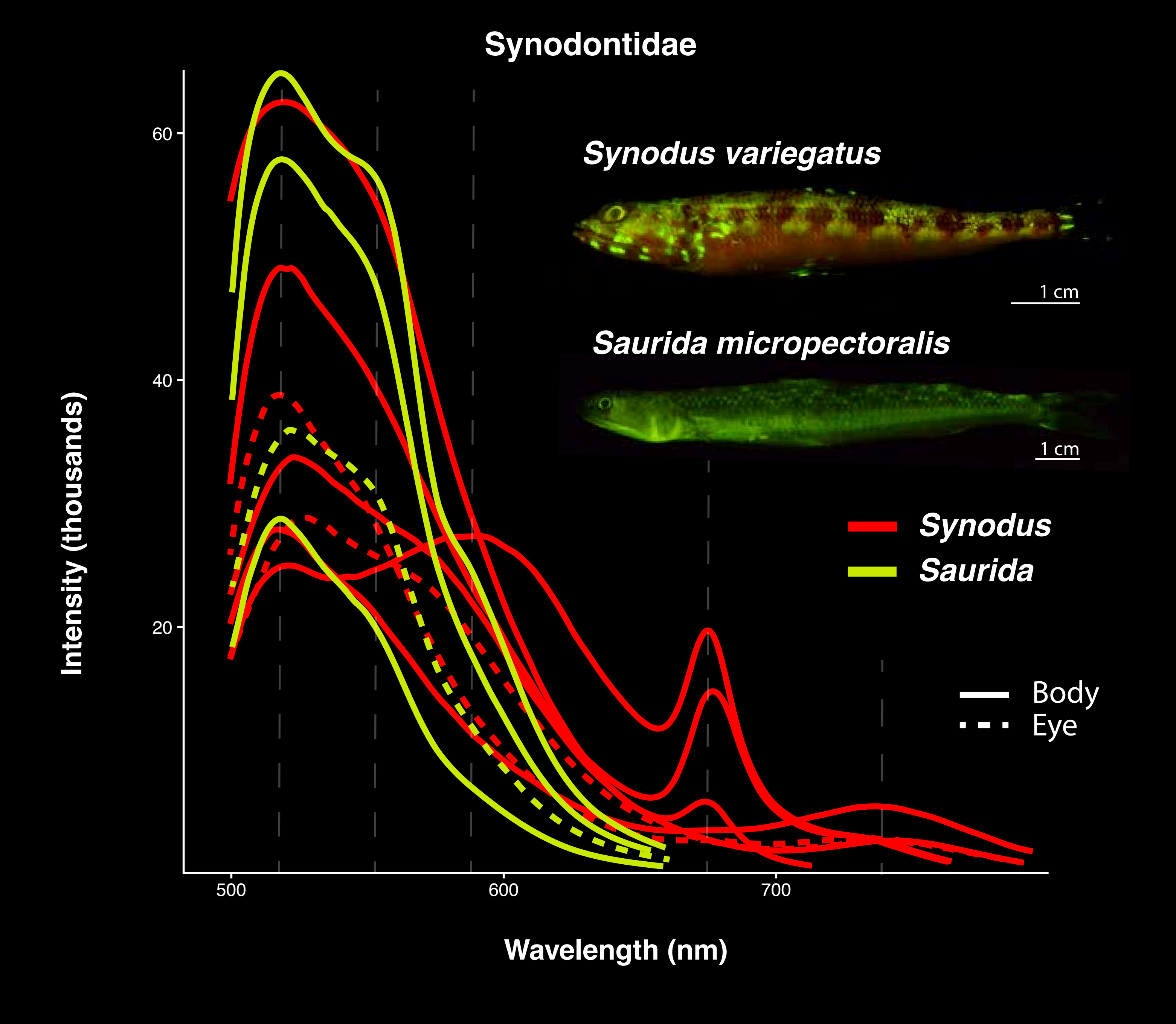
Synodontidae
Synodus variegatus
1 cm
Saurida micropectoralis
1 cm
Synodus
Saurida
Body
Eye
Intensity (thousands)
60
40
20
500
600
700
Wavelength (nm)
