# Supplementary material for: Marine fishes exhibit exceptional variation in biofluorescent emission spectra
Source: PLoS One. 2025 Jun 16;20(6):e0316789. doi: 10.1371/journal.pone.0316789 (PMC12169565; doi:10.1371/journal.pone.0316789)
Supplement: S3 Fig — (DOCX) [file pone.0316789.s003.docx]

**S24:**

Spectrophotometer Setup and Fluorescence Excitation Lighting:

For the fluorescent spectra readings, we either used two homemade royal blue collimated lights (490 nm +/- 5 nm) or two Sola NightSea lights set on full power and placed about 6-8 inches from the specimen at 45 degree angles to specimen (in a rostral and caudal orientation to the specimen). The two Sola Nightsea lights were set to maximum output in flood mode, which translates to 3000mW per light (450-460 nm; see spectral emission curve below). The two homemade royal blue LED light arrays utilize Omega Optical 490ASP excitation filters and output 12000mW each at a wavelength of 490 nm (+/- 5 nm). Technical specifications for all excitation light sources and excitation filters listed above, as well as for the Ocean Optics Ocean Optics USB2000+ portable spectrophotometer and hand-held fiber optic probe (Ocean Optics ZFQ-12135) used are available below.

Fluorescence Specimen Imaging:

For fluorescent imaging, we used a small photographic tank filled with seawater. Specimens were placed in the tank and gently held flat against a thin glass front. Fluorescence excitation was achieved using two Nikon SB910 Speedlights mounted on light stands and placed approximately 2 feet from the tank at 45-degree angles to the specimen (in rostral and caudal orientation to the specimen). The Nikon flashes were set on TTL, which does not allow one to determine power/intensity (see Nikon SB910 user manual). The distance between the camera lens and the species imaged depended on the lens focal length and the size of the specimen imaged (i.e., minimum working distance of the lens used either a Nikon 105mm macro or Sony 90mm macro). This distance was adjusted accordingly based on size of the specimen being imaged to fill the frame as much as possible. A Semrock EdgeBasic 514 nm long-pass filter was used to image all specimens except when it is specified that a Semrock 561 nm long-pass filter was used. Detailed imaging specifications for each fluorescent specimen shown in the Figures and supplementary materials (S4-S23) are presented below, as are technical specifications for both Semrock long-pass filters.

| **Figure** | **Family** | **Species** | **Focal Length (mm)** | **Exposure (seconds)** | **ISO** | **F-stop** |
| --- | --- | --- | --- | --- | --- | --- |
| Fig. S19 | Antennariidae | *Antennatus rosaceus* | 105 | 1/125 | 1000 | 5 |
| Fig. 2P, S9 | Aulostomidae | *Aulostomus chinensis* | 60 | 1/200 | 800 | 9 |
| Fig. 2E, S18 (bottom) | Blenniidae | *Ecsenius axelrodi* | 105 | 1/200 | 800 | 5 |
| Fig. S18 (top) | Blenniidae | *Ecsenius axelrodi* | 105 | 1/200 | 125 | 40 |
| Fig. 2G, 3E, S21 | Bothidae | *Japonolaeops dentatus* | 105 | 1/80 | 6400 | 8 |
| Fig. 2F, 3E, S21 | Bothidae | *Engyprosopon mozambiqense* | 90 | 1/20 | 800 | 5.6 |
| Fig. S8 | Cepolidae | *Cepola schlegelii* | 105 | 1/125 | 6400 | 8 |
| Fig. 2Q, S4 | Chlopsidae | *Kaupichthys diodontus* | 105 | 1/250 | 500 | 6.3 |
| Fig. 2H, S20 | Cynoglossidae | *Cynoglossus microlepis* | 105 | 1/80 | 6400 | 8 |
| Fig. 2A, 3B, S15 | Gobiidae | *Eviota prasites* | 105 | 1/250 | 250 | 9 |
| Fig. 3B, S15 | Gobiidae | *Pleurosicya micheli* | 105 | 1/250 | 250 | 9 |
| Fig. 2B, 3B, S15 | Gobiidae | *Trimma fangi* | 105 | 1/250 | 640 | 4 |
| Fig. 2J, 3A, S10 | Labridae | *Cheilinus oxycephalus* | 105 | 1/200 | 500 | 8 |
| Fig. 2K, 3A, S10 | Labridae | *Pseudocheilinus evanidus* | 90 | 1/250 | 125 | 6.3 |
| Fig. S11 | Liparidae | *Liparis gibbus* | See Gruber and Sparks 2021 | | | |
| Fig. S12 | Mullidae | *Upeneus sundaicus* | 105 | 1/100 | 6400 | 8 |
| Fig. 2I, 5D, S5 | Muraenidae | *Gymnothorax zonipectis* | 90 | 1/250 | 1250 | 10 |
| Fig. S13 | Nemipteridae | *Nemipterus tambuloides* | 105 | 1/100 | 6400 | 8 |
| Fig. 3D, S16 | Oxudercidae | *Gnatholepis anjerensis* | 105 | 1/250 | 640 | 6.3 |
| Fig. 2N, 3C, 5B, S14 | Scorpaenidae | *Taenianotus triacanthus* | 105 | 1/250 | 1000 | 4.5 |
| Fig. 2O, 3C, S14 | Scorpaenidae | *Sebastapistes fowleri* | 105 | 1/250 | 640 | 7.1 |
| Fig. S22 | Soleidae | *Heteromycteris hartzfeldi* | 105 | 1/80 | 6400 | 8 |
| Fig. 3F, S7, S23 | Synodontidae | *Synodus variegatus* | 90 | 1/250 | 640 | 9 |
| Fig. S7, S23 | Synodontidae | *Synodus binotatus* | 105 | 1/250 | 80 | 4 |
| Fig. S6, S23 | Synodontidae | *Saurida tumbil* | 105 | 1/80 | 6400 | 8 |
| Fig. 2L, 3F, S6, S23 | Synodontidae | Saurida micropectoralis | 105 | 1/80 | 6400 | 8 |
| Fig. 2C, S17 | Tripterygiidae | *Enneapterygius niger* | 105 | 1/200 | 200 | 6.3 |
| Fig. 2D, S17, 5A | Tripterygiidae | *Helcogramma striata* | 105 | 1/250 | 500 | 5 |

**Camera settings utilized for all fluorescent images of specimens presented in Figures 2,3, and 5, and Figures S4-S23).**


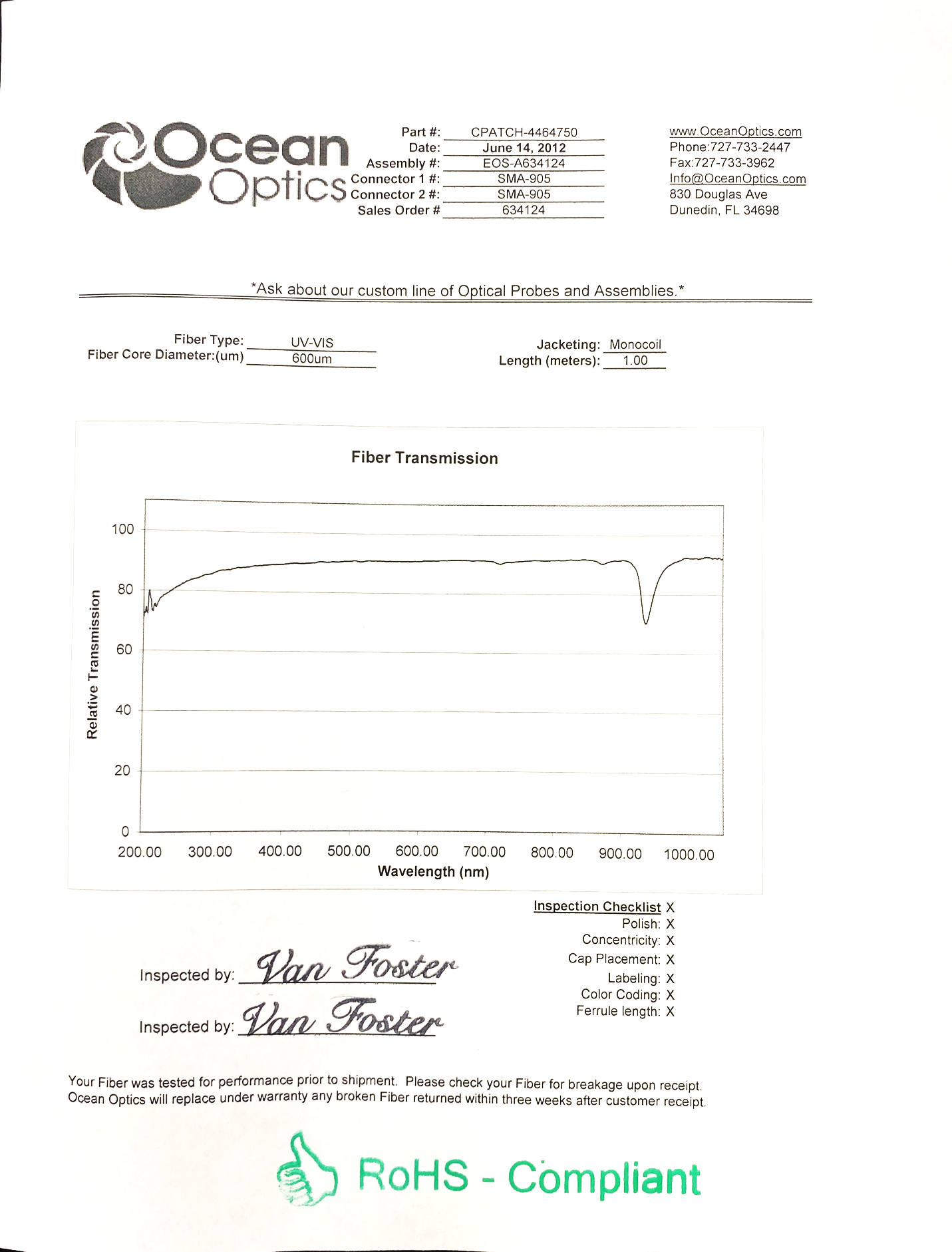


**Ocean Optics ZFQ-12135 spectrophotometer probe specification sheet.**


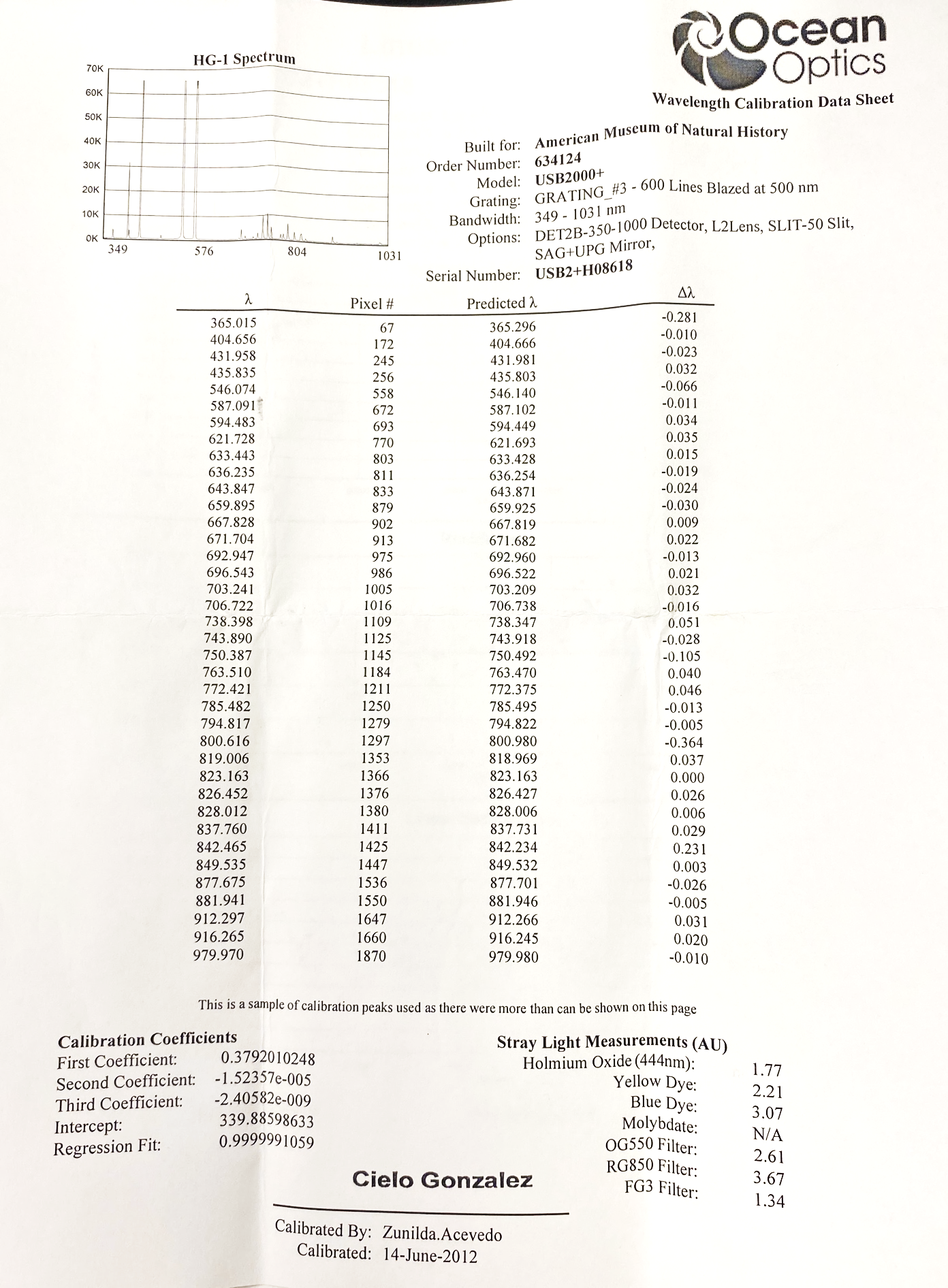


**Ocean Optics USB2000+ spectrophotometer specification sheet (page 1 of 2).**


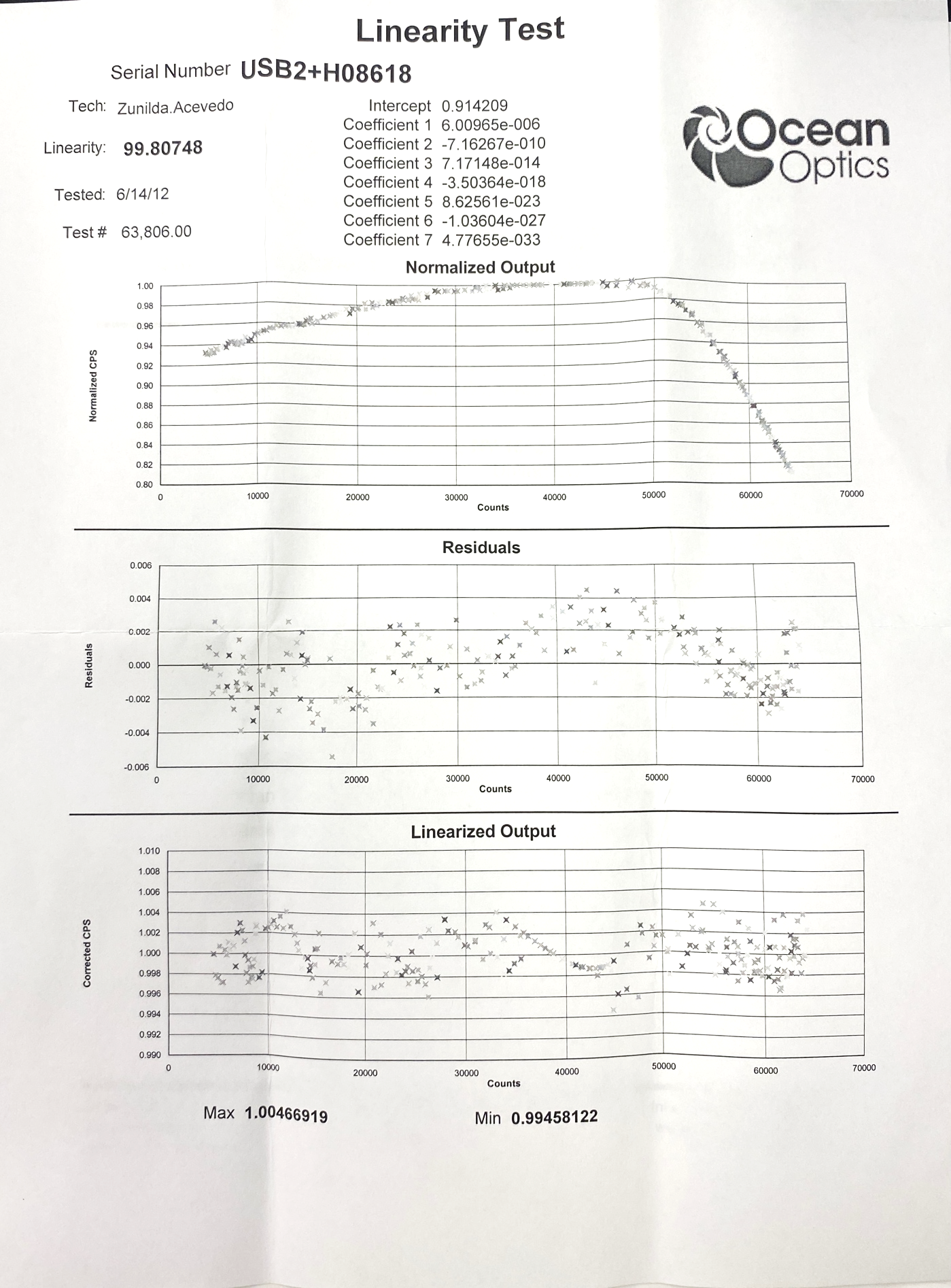


**Ocean Optics USB2000+ spectrophotometer specification sheet (page 2 of 2).**

**
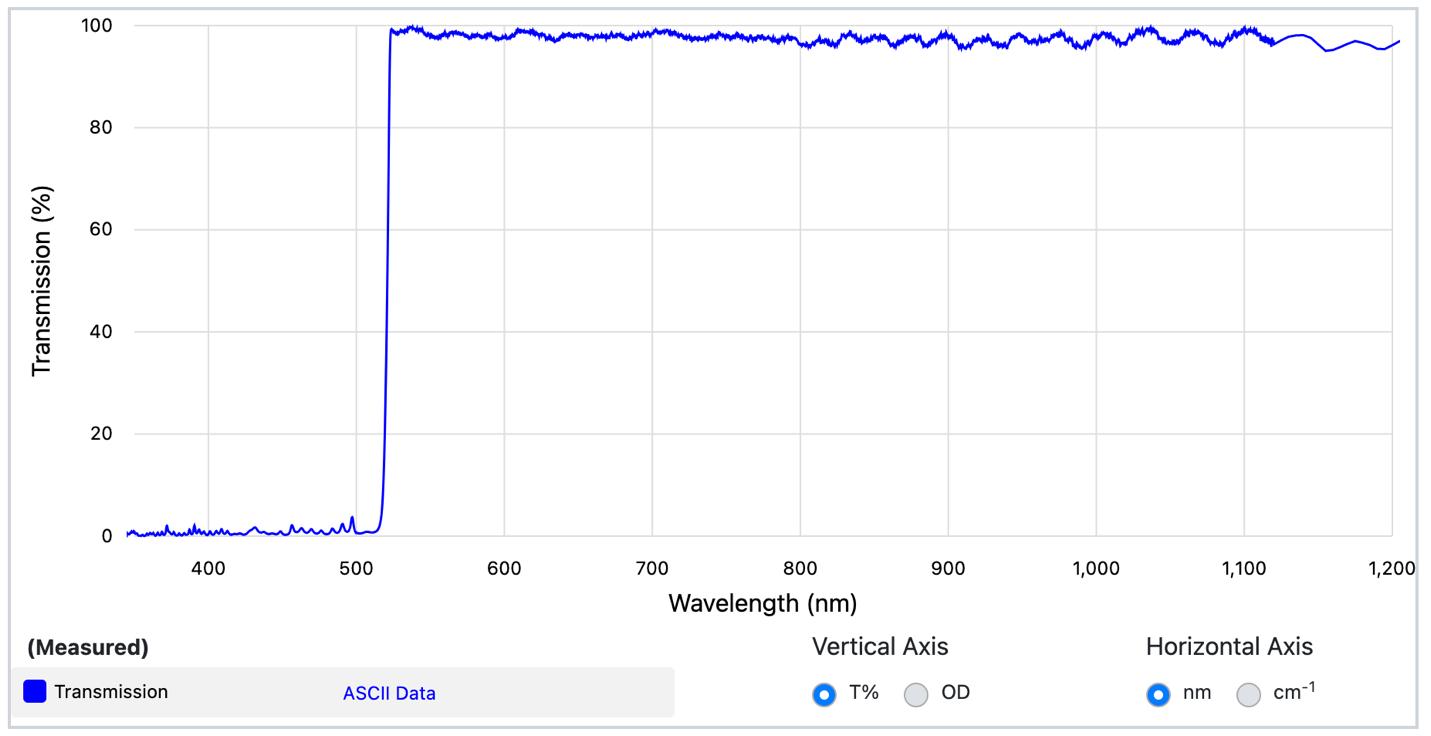
**

**Semrock EdgeBasic 514 nm long-pass filter spectral transmission curve. More information is available at** [www.avr-optics.com](http://www.avr-optics.com).

**
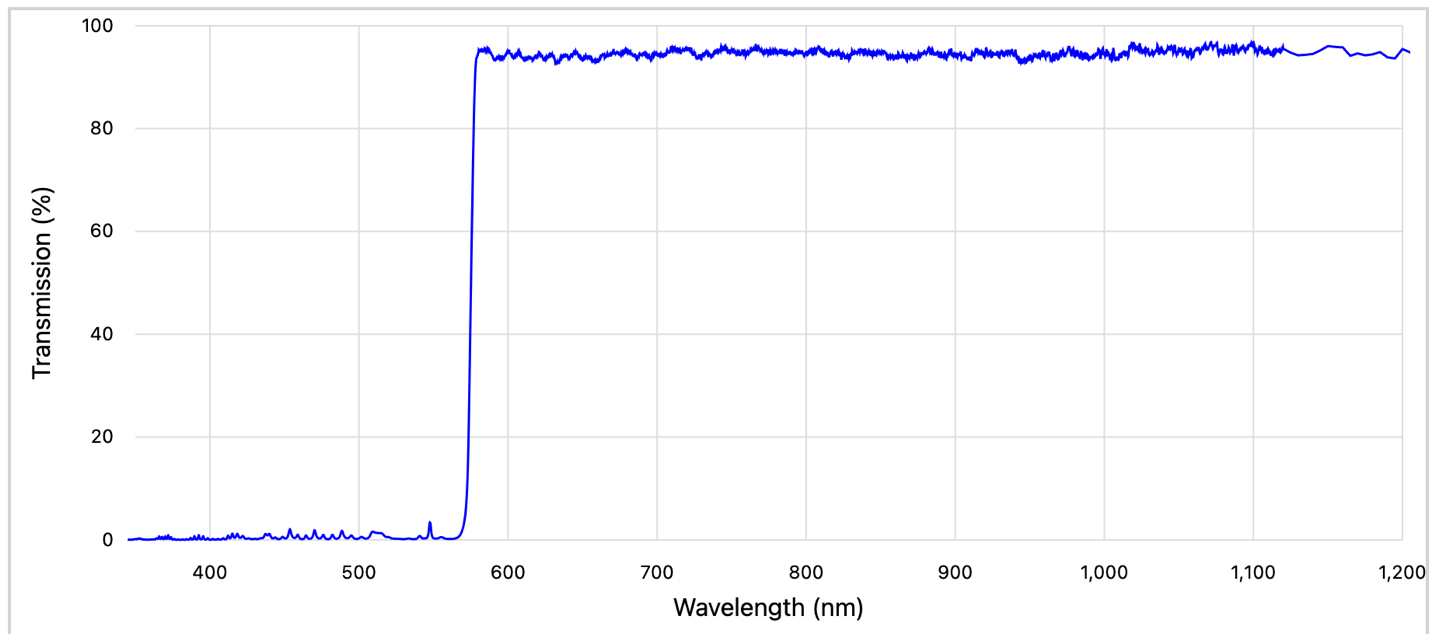
**

**Semrock EdgeBasic 561 nm long-pass filter spectral transmission curve. Filter used to visualize emitted yellow through red fluorescent wavelengths in specimens with both green and red fluorescence. More information is available at** [www.avr-optics.com](http://www.avr-optics.com).


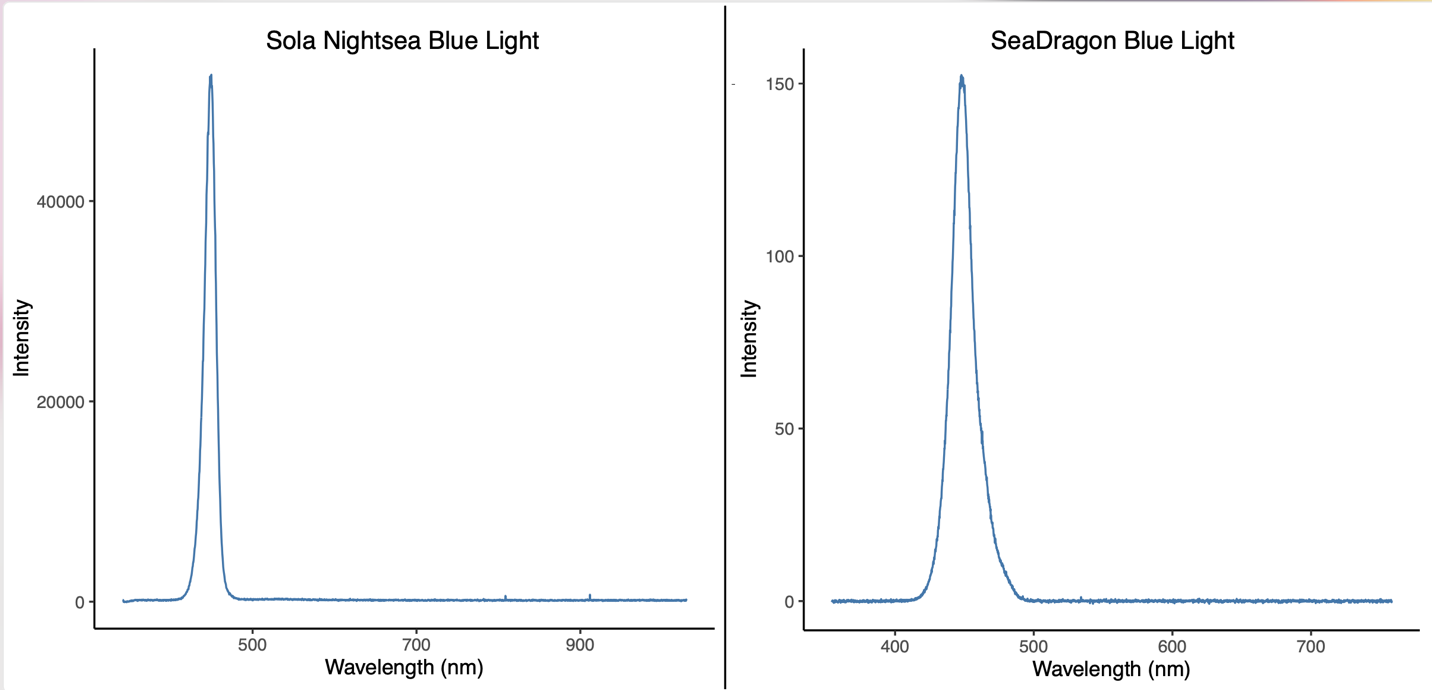


**Spectral emission curve for Sola NightSea blue excitation lights utilized to record fluorescent emission spectra.**

**
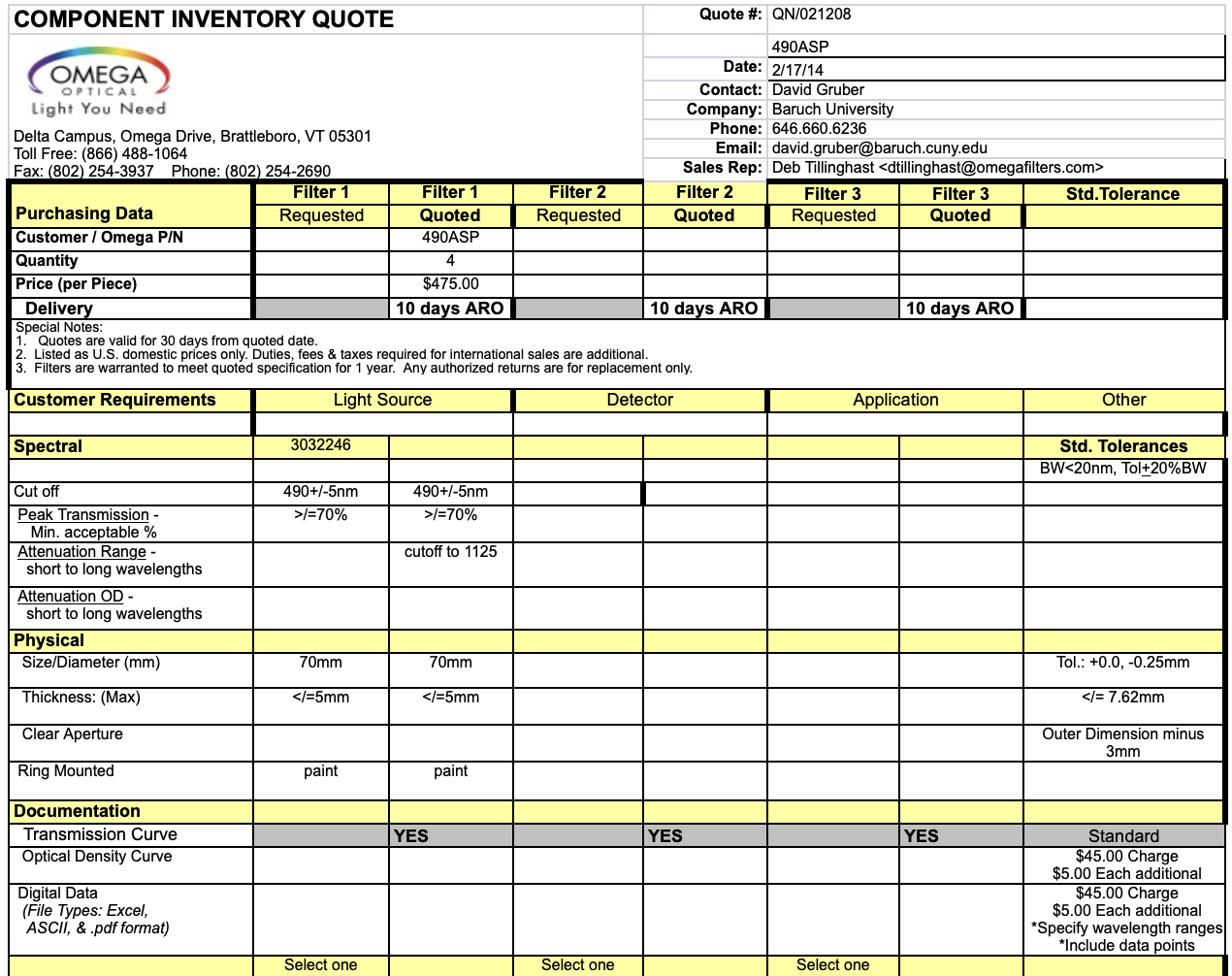
**

**Technical specifications for Omega Optical blue excitation filters (490 +/- 5 nm) utilized for both fluorescence imaging and to record fluorescent emission spectra.**
